# Caring for the patient, caring for the record: an ethnographic study of ‘back office’ work in upholding quality of care in general practice

**DOI:** 10.1186/s12913-015-0774-7

**Published:** 2015-04-23

**Authors:** Deborah Swinglehurst, Trisha Greenhalgh

**Affiliations:** 1 Centre for Primary Care and Public Health, Barts and The London School of Medicine and Dentistry, Queen Mary University of London, Yvonne Carter Building, 58 Turner Street, London, E1 2AB UK; 2 Nuffield Department of Primary Care Health Sciences, New Radcliffe House, Radcliffe Observatory Quarter, Woodstock Road, Oxford, OX2 6GG UK

**Keywords:** Electronic patient records, Ethnography, Quality of care, Coding, Classification, Administration

## Abstract

**Background:**

The quality of information recorded about patient care is considered key to improving the overall quality, safety and efficiency of patient care. Assigning codes to patients’ records is an important aspect of this documentation. Current interest in large datasets in which individual patient data are collated (e.g. proposed NHS care.data project) pays little attention to the details of how ‘data’ get onto the record. This paper explores the work of summarising and coding records, focusing on ‘back office’ practices, identifying contributors and barriers to quality of care.

**Methods:**

Ethnographic observation (187 hours) of clinical, management and administrative staff in two UK general practices with contrasting organisational characteristics. This involved observation of working practices, including shadowing, recording detailed field notes, naturalistic interviews and analysis of key documents relating to summarising and coding. Ethnographic analysis drew on key sensitizing concepts to build a ‘thick description’ of coding practices, drawing these together in a narrative synthesis.

**Results:**

Coding and summarising electronic patient records is complex work. It depends crucially on nuanced judgements made by administrators who combine their understanding of: clinical diagnostics; classification systems; how healthcare is organised; particular working practices of individual colleagues; current health policy. Working with imperfect classification systems, diagnostic uncertainty and a range of local practical constraints, they manage a moral tension between their idealised aspiration of a ‘gold standard’ record and a pragmatic recognition that this is rarely achievable in practice. Adopting a range of practical workarounds, administrators position themselves as both formally accountable to their employers (general practitioners), and informally accountability to individual patients, in a coding process which is shaped not only by the ‘facts’ of the case, but by ongoing working relationships which are co-constructed alongside the patient’s summary.

**Conclusion:**

Data coding is usually conceptualised as either a technical task, or as mundane, routine work, and usually remains invisible. This study offers a characterisation of coding as a socially complex site of moral work through which new lines of accountability are enacted in the workplace, and casts new light on the meaning of coded data as conceptualised in the ‘quality of care’ discourse.

**Electronic supplementary material:**

The online version of this article (doi:10.1186/s12913-015-0774-7) contains supplementary material, which is available to authorized users.

## Background

The quality and integrity of information recorded about patient care is widely considered as key to improving the quality, safety and efficiency of patient care and to informing high quality health service planning [1]. ‘Data quality’ hinges crucially on assigning codes to patient records and ensuring that data are comprehensive, accurate, complete, and ‘fit for purpose’, a key advantage of coded data being their availability to institutional audit and research [1-4]. Although there is no single established method for ascertaining the ‘quality’ of this coding in terms of completeness or correctness [5], the idea that high quality coding is achievable and that “*better information means better care*” goes largely unquestioned [6]. Identifying barriers to clinical coding is regarded as the first step to addressing the problem of inadequate patient records [7,8]. For example, a document entitled ‘*Good Practice Guidelines for GP electronic patient records*’, now in its fourth version, asserts (page 11):

*There is a need to develop new guidance in areas such as high quality clinical records and data quality to facilitate records sharing, inter-operability and communication within a clinical safety framework* [9].

The UK’s controversial National Programme for IT, with its aspiration to a fully inter-operable networked electronic record accessible from all points of care, was formally abolished from 2010 [10]. But the political zeal for the principle of data capture and standardised comparative datasets remains strong: “*recording data once and using it in many ways*” [11]. This principle underpins current interest in large datasets in health care initiatives such as the NHS care.data project which seeks not only to ‘*improve the quality of care for all*’ but to enable the public to ‘*hold the NHS to account and ensure that any unacceptable standards of care are identified as quickly as possible*’ [6]. Well established programmes such as the UK Quality and Outcomes Framework (QOF) which (since 2004) rewards UK general practitioners financially for demonstrating performance against a range of clinical and organisational indicators (comprising 30% of practice remuneration) depend on assiduous data coding, and on a taken-for-granted assumption that the quality of care can be inferred from the quality of these data (for a critique of this assumption see [12] *[within this BMC special collection –please insert citation]*). The emerging academic discipline of primary care informatics sees the scientific study of how to harness these ‘routinely collected’ data as one of its main concerns [4]. In the setting of UK general practice, the Read code system is the standard terminology system used for coding and is compiled, maintained and updated by the Health and Social Care Information Centre [13].

Surprisingly absent from the academic literature or policy documentation around ‘routinely collected’ coded data is any account of how these data emerge from practice, or what constitutes the ‘work’ of producing the data which underpins the normative discourse of ‘quality of care’. It is nevertheless well recognised that the act of coding medical diagnoses imposes a bio-medical model and tends to carry unwarranted assertions about diagnostic certainty, when disciplines such as general practice are characterised by uncertainty and constructed through complex social interactions which are difficult to pin down [7]. Within the informatics literature, barriers to recording structured coded information tend to be broadly described as either ‘technical’ or ‘socio-cultural’ [7]. An imagined ‘ideal’ future situation in which these barriers will be circumvented and a highly standardised structured electronic record - unhampered by imperfect data - will emerge, which reflects and informs quality of care, is a prominent current policy aspiration [6,7,11,14].

Alongside this mainstream understanding of coding and its contribution to care is a sociologically informed literature in which the tension inherent in what is called the *rationality-reality* gap [15] or the *fatal paradox* [16] between the nature of health care work and the standardisation of this work through formalising technologies and procedures (such as coding) is recognised as a site of complex moral work and worthy of investigation in its own right [17,18]. In Bowker and Star’s seminal work on classification, for example, which includes tracing the history and politics of the ICD-10 (International Classification of Diseases) we are reminded that “*each standard and each category valorizes some point of view and silences another*” (page 5) [17], and that the work of those who do the classifying is (therefore) highly consequential.

Berg has argued strongly against becoming entrenched in the duality, or opposition, of the formal and the informal, or pitching the ‘*complexity of medical work*’ against ‘*impoverished representations*’ of it, instead suggesting it is more productive to focus on *practices* and to consider the ways in which skilful human work bridges the rationality-reality gap [19,20]. Suchman who conducted ethnographic research exploring the mediating work of document coders within a legal department, has highlighted this work as one of interpretation and judgment, arguing for the importance of looking for and appreciating ‘invisible work’ – the work of those whose role is to make ‘usable’ *and* ‘useful’ (i.e. to recontextualise) in local sites of practice those technologies which may have been designed at a distance [21,22]. Suchman identified the work of coding documents as an interweaving of tedious activity, mindful judgment and practical reasoning. Litigation support staff were found to be continuously experimenting with alternative strategies for coding documents, engaged in reflecting on and redesigning their own work practices; work that was typically characterised in the law firm as ‘routine’ – and assumed to be a relatively unskilled, mindless activity – was found to involve considerable ‘knowledge work’ [22,23].

Attempts to standardise clinical terminology predate the widespread adoption of electronic patient records (EPRs) in general practice. However, the ascendancy of evidence-based-medicine, the introduction of the QOF and an increasing emphasis on accountability in the public sector *coupled with* the widespread use of EPRs has changed what is made possible through attention to such classification, and has resulted in substantial changes to working practices and organisational routines [24]. Attention to appropriate coding of patients’ electronic records has become an increasingly important concern.

In this research we adopted a socio-technical approach, an important premise of this perspective being that information technologies, such as the EPR, cannot be meaningfully studied in isolation from the social situations in which they are used (or, sometimes, in which they are not used, or are ‘worked around’) and that technologies shape – and are to some extent shaped by – human action [19,20,25]. Our focus in this paper is our study of the socio-technical practices of ‘back office’ administrative staff as they used EPRs in the conduct of their routine day-to-day work. Two particular aspects of this ‘routine’ administrative work which emerged as critically important to notions of ‘quality’ in the delivery of care in general practice were the summarisation and coding of patients’ medical records in the EPR. In this paper we present detailed ethnographic analysis of this work and identify the coding of a patient’s EPR as an integral part of organisational life and one around which relationships are negotiated and new lines of accountability constructed. The coded record is not simply the product of a technical process, or the work of an individual, but is the result of considerable and collaboratively organised ‘care’, directed at balancing the needs of the patient, the needs of the institution and a complex range of social, technical and political contingencies, as the coder strikes a balance between the ‘ideal’ record and that which is achievable within the constraints of normal day-to-day organisational life.

## Methods

The study was part of the Healthcare Electronic Records in Organisations study, funded by the UK Medical Research Council. NHS ethics and governance approval was granted (Thames Valley multicentre research ethics committee approval 06/MRE 12/81). The background and protocol for the study have been previously published [26], along with a theoretical justification for the use of ethnographic approaches to study technologies-in-use in healthcare [25]. This part of the study was conducted in two urban UK GP surgeries (pseudonymised Clover and Beech), to which access was gained through general practitioners inside the organisations - a legitimate approach in ethnographic work [27] and one which favours what Stake calls ‘opportunity to learn’, over concerns about ‘typicality’ [28]. Hammersley and Atkinson point out that “*Gaining access is a thoroughly practical issue…it involves drawing on interpersonal resources and strategies that we all tend to develop in dealing with everyday life*” (page 54) [29]. As a principle we agreed only to study organisations in which every member of staff had given informed written consent for the research to be undertaken in their organisation. A tiered approach to consent ensured that staff members could decline being directly ‘shadowed’ as individuals and/or decline consent to being interviewed or quoted in published work. But all members of staff had given explicit consent to our presence in the organisations as observing researchers.

The surgeries served mixed patient populations of approximately 11800 (Clover) and 12600 (Beech) respectively at the time of data collection (2007–2008). Both were providing services from converted houses in residential areas, using a clinical system called EMIS-LV which was the most widely used clinical system in the UK at the time of the study. Electronic records were used in both sites for booking appointments, clinical record keeping, prescribing and a range of ongoing institutional audits and financial activity. Clinicians, administrative and support staff in both surgeries had been using the EPR for several years and as such were accustomed to its operation. Clover and Beech scored highly in the Quality and Outcomes Framework.

DS conducted 187 hours of ethnographic observation (distributed over four months in each of the two practices) during normal working hours, in sessions which usually lasted 3–5 hours. Across the two study sites, DS observed 15 doctors; 9 nurses; 3 healthcare assistants; 9 administrative/secretarial staff; 2 information managers; 2 practice managers; 16 receptionists. This included what Werner and Schoepfle refer to as “*lurking and soaking*”, shadowing of individual staff, recording of detailed field notes and opportunistic naturalistic interviews (“*Would you be happy to talk me through what you are doing?*”). Workers are typically unable to describe what they do unless they are doing it and this approach was deemed more sensitive to the particularities of local contingencies and contexts than formal interviews [30]. We advised staff that we were keen to study routine day-to-day working practices and that there was no attempt being made to assess individuals’ performance. We also collected local documents (such as protocols) relating to summarising and coding patients’ records. Field notes made on site were typed up on the same day, anonymised, shared between the researchers, and annotated with observational and theoretical notes [31]. We sought to build a ‘thick description’ [32] of organisational life from the bottom up, adopting an interpretive perspective of organisation - a perspective which considers that ‘organisational culture’ is not a ‘given’ but is accomplished through recurring patterns of relationships and co-constructed meanings and actions [33].

## Results and discussion

In this section we will begin by briefly describing some broad characteristics of the two study sites as context, with particular attention to their approach to new technologies and technological change. We will then draw on two extensive extracts of ethnographic field notes as a basis for examining coding and summarising practices. These field notes are presented in an Additional file.

### Characteristics of the two study sites

Although the brief description of the two study sites in the Methods section would suggest two similar surgeries there were important differences. Both surgeries had the same number of GP Principals and similar patient list size, but Beech surgery had twice as many nurses as Clover (the manager at Clover joked “*We are not paper light but we are nurse light*” on an introductory tour of the practice). Clover employed almost three times as many administrative/secretarial staff as Beech. Each practice employed someone whose main role was in Information Management and Technology (IM + T). In Beech this was regarded as a senior administrative role and was occupied by a female member of staff who had started working as a junior administrator in the practice and had gained seniority over time. In Clover the person in this role was also ‘Assistant Manager’ with some line management responsibilities for the administrative staff that he oversaw in a shared office. He had a background in non-NHS IM + T work prior to joining the practice several years earlier. The female practice manager at Beech had been there for over twenty years, starting her career as a medical secretary. The male manager at Clover had joined nine years ago, following a previous career in finance. Both practices were perceived by their staff as very good, congenial places to work, committed to high quality patient care.

Broadly, organisational ethos at Beech surgery was that of the ‘traditional family doctor’ with an emphasis on continuity of care and interpersonal relationships. The management valued teamwork and interpersonal relationships over bureaucratic procedures, the word ‘team’ in organisation-wide use as a descriptor of the surgery. Working relationships between clinical and non-clinical staff were close; they regularly engaged in *ad hoc* unscheduled interaction in cramped shared working spaces such as the reception area. The workforce was stable and there was much informal sharing of ‘know-how’ and – at least among non-clinical staff – a significant amount of cross-over of roles (both formal and informal). For example, all members of the administrative staff were ‘reception-trained’; the practice manager would provide reception cover if necessary, and practice nurses were seen greeting patients at the reception desk if the queue was long. Differences in working practices between clinicians were widely acknowledged to exist, but this was not *usually* framed as a problem which required an organisational ‘fix’ but was instead tolerated and accepted as an inevitable part of practice life. In general, the doctors and management at Beech adopted a cautious approach to new technologies; they did not regard themselves as technology innovators but were ready to adopt new technologies if convinced of the benefits to patient care.

Clover adopted a ‘modern business’ ethos with greater emphasis on uniformity, standards, protocols and ‘customer care’ practices. The management style was (relatively) ‘top down’ with stricter observation of roles, clear hierarchies and relatively higher levels of bureaucracy. Documents, written policies and protocols were highly valued by management and staff alike, who routinely referred to Clover as “*the business*” (e.g. “*this side of the business*”, “*that side of the business*”, “*the needs of the business*”). There had been a higher turnover of non-GP staff, almost all of whom had joined the practice within the last five years. Knowledge transfer was generally articulated in terms of ‘training’ and was usually viewed as a separate activity to ‘work’. However, within each staff group (e.g. administrators or secretaries) there was much informal helping and mutual awareness of each others’ working activities. As in Beech, there were disparate working practices between clinicians, but this was more readily identified by management (and administrators) as a problem demanding an organisational ‘fix’. That the doctors did not work in more similar ways was sometimes regarded as an impediment to progress. The management at Clover adopted new technologies readily and took pride in being ‘ahead of the game’ compared to other local practices with respect to IM + T.

During the four months of data collection at Clover, the computer server was replaced with a higher specification model; there were purchases of cordless telephone handsets for administrators, portable hard drives and digital recorders; a new networked electronic ‘panic alarm’ system was installed, and staff began using new software for transferring electronic records between GP practices – Clover was one of the first practices in the county to do so. Plans were afoot to install plasma screens in the waiting area with a touch screen for patients to use to ‘check in’; it was hoped that this would reduce receptionist hours, freeing time which could be used for more efficient coding of electronic records. Although there was broad agreement to these changes, enthusiasm was not unanimous. One GP lamented the loss of the “*last personal touch*” and another was concerned that his routine of going into the waiting room to call patients might be challenged under the new system. The pace of change was proving difficult for some staff. One secretary who retired (early) during the fieldwork after 22 years of service blamed the computer (specifically software called “Choose and Book”) for her departure (“*the computer got the better of me*”; “*the patients have always been my main concern here. I don’t know where patients are these days - lost under piles of paper and the Choose and Book system*”).

### Investigating the EPR ‘backstage’ at a pro-technology practice

In the remainder of this paper we will draw primarily on ethnographic observations from Clover surgery - which we identify as a ‘pro-technology’ surgery - to reflect on the relationships between use of the EPR, organisational practices of coding and summarising patient records and notions of ‘quality’ in health care. The term ‘backstage’ from Goffman [34] is used to describe settings that are not directly ‘patient-facing’, where work *with and about* patients (including their representations in texts and records) does not also involve *interacting with* patients directly. We adopt a socio-technical perspective, recognising that one cannot meaningfully study technologies in isolation from the social situations in which they are used (or not used, or worked around) and that technologies shape – and are to some extent shaped by – human action [19,20,25].

We select Clover as an example of what Mitchell calls a ‘telling case’ (page 239) [35], our explicit intention being to explore what is accomplished through coding and summarising records in a surgery that readily embraces technologies such as the EPR. Coding, in this context, is the ongoing process of assigning Read codes to patients’ records (e.g. important diagnoses, investigations) and occurs both within consultations (when clinicians do the coding) and in clinical and administrative areas on receipt of important communications about patients (e.g. discharge summaries, letters from outpatient clinics). Summarising is a related process of creating a succinct list of the most salient Read codes in one part of the record (‘the summary’) and typically occurs when a new patient joins the practice list, or as part of ongoing processes of improving information management (at the time of data collection there were QOF points available for ‘summarised’ records).

#### Care and ‘aftercare’ for the EPR: summarising the electronic patient record

At Clover surgery, the summarisation and Read coding of patient records was a high priority activity. Although some coding was done in clinical consultations (especially by nurses doing chronic disease management reviews) [24], much of it occurred in the backstage regions of the surgery and was done by administrators, with guidance from doctors. Four members of administrative staff were involved, and for two of them it was their main administrative role. There was a fourteen page practice protocol on summarising medical records which was in its second version. It opened with the words:

***AIM:****The aim of summarising patients’ notes is to be able to easily access the past and present medical history of the patient via the computer screen. Using the protocol, information from patients’ notes is entered on screen using agreed Read codes. This enables future accurate auditing to be undertaken* [Reproduced from practice protocol].

The summarisation protocol went on to explain the procedure for sorting contents of the medical record and creating the summary; a list of the types of information which should be added; guidance on how to categorise summary data (as ‘active’ or ‘significant’); three pages of “Medical Problems and Read codes” (taken from a document supplied by the local Primary Care Trust) and a page of “Common Abbreviations”. The aim of summarising records was thus described in technological and institutional terms - the ease with which information can be accessed from the computer screen, with a particular reference to enabling institutional audit. The individual patient’s history is juxtaposed with the needs of the institution for ‘auditable’ data. The implicit assumption regarding ‘accuracy’ of this account is something we had observed in detailed micro-analysis of clinical consultations (published elsewhere) there being a tendency for the EPR to become the authoritative source of the patient’s past medical history, even when the patient was co-present, sometimes offering alternative versions of events [36].

Summarising patients’ records was regarded by administrators as responsible work demanding concentration. One of the summarisers liked to get into the office at 7:30 a.m. so that she could start summarising while the office was quiet and relatively free from distractions. Frequent reference was made (by administrators, manager, information manager and clinicians) to the amount of time and ‘care’ that went into this task. The IM + T lead explained that other surgeries did not take as much care over coding and summarising as his own staff, and one of the GPs (who was relaying concerns around the implementation of the new electronic system for transfer of records) highlighted their close attention to detail: “*They [the enthusiasts for electronic transfer of records] underestimate the work that goes into record keeping…they think it just happens in the consultation but you only need to look at how much care goes into the records, by people like [name of summariser]*”. In the words of the retiring secretary “*At one time there was no need for all those admin people – but now there is a whole room of them – all because of electronic records*”.

Care was needed not only in creating the record but also in maintaining it - ‘aftercare’. Various terms were used for this activity: “*cleaning*”, “*feeding*”, “*cleansing*”, “*tidying*” and even “*computer toilet*”. In Beech surgery, one doctor often went in early for morning surgery to spend time “*cleaning up*” the records of patients he was about to see, removing “*clutter*” and, if possible, reducing the number of ‘problems’ listed on the summary screen (“*I can’t stand it when there are 24 active problems showing – I’ll tend to clean it up*”). A “*cluttered*” or “*clogged up*” summary screen was regarded by clinical and non-clinical staff in both surgeries as something to be avoided if possible, there appearing to be an informally shared understanding that there is a limit to the number of ‘problems’ it is reasonable for a patient to have documented, a limit which related in part to the material constraints of the technology around what could be viewed conveniently on a summary screen at one time. In one consultation we observed at Clover, an elderly man returned to his GP following an X-ray of his hands which had confirmed osteoarthritis (OA) as the cause of his aching thumbs and wrists. He had been prescribed anti-inflammatory medication and was enquiring about other treatment possibilities. After the patient left the GP looked at the patient’s summary screen and commented: “*No one’s put OA here as a problem… I don’t think I’m going to put it in…I just think he’s got a lot of diagnoses already*” suggesting he had reached this ill-defined limit. The diagnosis was entered as free text, but not Read coded or afforded the status of ‘Problem’ on the summary screen, making it more difficult to find in future consultations (and ‘invisible’ to any audit process).

In a specific example of ‘aftercare’ of the EPR, a doctor at Clover circulated an email to all staff explaining he was manually editing over 200 patients’ EPRs. Population Manager (software integral to the EMIS clinical system which searches daily for QOF-relevant Read codes and identifies patients with ‘missing’ data items) was identifying these patients as ‘*severely mentally ill and needing reviews*’ (a QOF requirement) although it had become clear to him (on closer inspection of these patients’ EPRs) that this was because of the abundance of Read codes for ‘*recurrent depression*’ and ‘*endogenous depression*’. Although these codes may have been ‘fit for purpose’ at data entry, they were now being captured in automatic daily audits of the practice population for the purposes of a QOF target intended only for those with ‘*severe and enduring mental illness*’ (e.g. psychoses). This additional work of editing the EPRs ensured the practice was not penalised financially for failing to offer detailed health checks to patients whose clinicians recognised did not fulfil the criterion ‘severe enduring’ mental illness.

That the process of creating and maintaining summaries was resource intensive was well recognised and was specifically highlighted in one of Clover’s newsletters for patients, at the end of a section detailing areas of expenditure in the practice. It read:

*Of course our main costs in keeping up to date are the employment of staff in updating our records and summarising our notes* [Reproduced from Clover Newsletter].

Not only does this draw attention to the financial costs of summarising records, but it also constructs this activity as centrally important to ‘keeping up to date’ and an inevitable part of the modern GP surgery.

In Additional file 1: Box 1 we present an extract of ethnographic field notes to consider the ‘care’ that goes into summarising a patient’s record.

To get her job done (Additional file 1: Box 1) Amy drew on her knowledge of a wide range of formal and informal resources. They included texts (e.g. notebook, dictionary, Internet, different parts of the patient’s existing records, summarising protocol, her ‘memory’ cards); specific technologies (Read codes, electronic messaging system); current health policy (QOF) and locally accepted procedures; the expertises of her coding colleague and a GP. She mapped her understanding of what Mr Oliver had ‘*wrong with him*’ against local practice policy about how problems should be categorised, her understanding of QOF, and unofficial norms about whom to seek help from (and in what order). Drawing on her working experience of the Read code formulary and her understanding of the relative importance of particular diagnoses she made judgements regarding what constitutes a reasonable amount of time and effort to spend seeking out particular codes and resolving particular ‘diagnostic’ dilemmas. For example, she assigned greater importance to resolving the conundrum around this patient’s possible atrial fibrillation/postural hypotension than to fine tuning the coding of the patient’s testicular operation. She also made personal notes (‘memory’ cards) about Read coding as part of an ongoing process of reflective learning.

Amy explained that she liked to summarise records as she would like her own record summarised. Another said that she felt strongly that when she was coding and summarising records she was working *for* the patients, whilst recognising that ‘officially’ she was working for the GPs. When summaries were received from other surgeries, they would start the process of summarising again, perceiving that summaries generated elsewhere were poorer in quality, missing important items and generally not to be trusted (“*there are summaries and there are summaries*”). The recent decision to start using GP2GP software for transfer of electronic records between practices had done nothing to change this. Summarisers continued to ‘start over’ with summarising, placing higher value on their own summarising judgements than those of an unknown (and anonymous) coder from a distant practice.

Local ownership of the summarising process was important to the summarisers’ sense of professional identity. They took immense pride in identifying items of history that previous summarisers had missed, ‘improving’ summaries by extending them and annotating existing Read codes with qualifying free text. In the administrative areas of the practice, the patient’s data and information in the EPR is the substrate from which administrators can carefully and creatively ‘mould the EPR into shape’ for its new organisational context. Through this process of building, extending and ‘improving’ the EPR, administrators contributed to the construction and redefinition of this new information context, building their own identities as ‘good’ summarisers as they went along.

The sense of being informally accountable to patients and officially accountable to GPs and the wider institution is something that we have also reported in the work of GP receptionists [37] and was sometimes a source of tension. Although in their role as summarisers they didn’t meet patients in person, working only with the patients’ records (Robinson has coined the term ‘*patient inscribed*’) [38], administrators were constantly making balancing judgements about the role of the EPR in supporting individual patient care (‘*patient as person*’), alongside broader institutional demands for record keeping. They had built a strong professional sense of working *for* the patient within these institutional constraints. For the summarisers, a ‘good’ summary was one which was extensive, thorough and complete, in which *nothing had been missed out*. The GPs, by contrast, had more modest ambitions. Administrators suspected (rightly) that some of the GPs felt they spent too much time on their summaries, producing summaries which were too detailed, when something more ‘basic’ would do. This inter-professional contest over what constituted a high quality summary was one we witnessed on numerous occasions. Even within summarisers’ own practice there was a constant tension between framing the practice of Read coding as highly responsible work (“*I think it’s really serious. I mean if I get it wrong it could have serious consequences*”) and a recognition that there are limits to how long one can spend searching for a suitable Read code that may or may not exist (“*that’ll have to do*”).

Until just before our fieldwork began, the GPs’ role in summarisation at Clover Surgery had been limited to making judgements about whether and how to Read code ‘new’ diagnoses which were relevant to the QOF (with implications for targets, QOF-related workload and practice funding). As much QOF performance was based on activity within the previous 15 months, this determined the definition of ‘new’ diagnoses. Summarisers would alert GPs to these potential diagnoses as they came across them, cognisant of the fact that some diagnoses (e.g. atrial fibrillation or diabetes), were more consequential than others (such as multiple sclerosis or osteoarthritis) in institutional terms, thus keeping the ‘institutional’ version of the patient in mind. Once certain Read coded diagnoses were entered into the EPR the patient became part of the ‘denominator population’ for a range of QOF targets, with commensurate demands on the organisation to meet these (or risk forfeiting financial incentives). Institutionally, higher stakes attached to these diagnoses than to other diagnoses which might be comparable, from the patient’s perspective, in terms of symptoms or impact on daily life. Accordingly, different levels of ‘care’ (delivered by different professionals) attached to different entries in the patient’s EPR in the ‘backstage’ regions of the practice, some entries (or potential entries) attracting greater scrutiny than others. This careful attention to codes (or diagnoses) which aligned with institutional goals (e.g. high QOF achievement) is something we also observed in ‘front stage’ regions of the clinic, such as nurse-led chronic disease consultations [24]. In the next section we consider the additional socio-cultural work that gets done as the inter-professional contest over what constituted a ‘quality’ summary played out in one particular aspect of the summarising routine in Clover surgery.

#### The constitution of professional hierarchies and local accountabilities

In recent months, GPs at Clover had started assisting more closely with the summarising process, the aim being that Clover would achieve a ‘higher percentage of notes summarised’ (itself a QOF target, and also a requirement of GP training practices). This highlighted some differences in the approaches taken by administrators and doctors and provided an opportunity for some interesting accountability work to be enacted.

One of the summarisers had distilled Clover’s official fourteen page summarisation protocol into a simpler one page document which was given to each GP, called “*Summarising of Patients’ notes – a short overview of what we currently do!*” Additional file 1: Box 2 shows some sections of this document (the italics are our own). Although introduced as a ‘*short overview of what we currently do*’ and incorporating a detailed list of what ‘*we include*’ the document is also replete with requests to doctors to do things in certain ways e.g. ‘*For each diagnosis please write the exact date of the 1st onset (not just the year please!’)*.

An understanding of this document (Additional file 1: Box 2) requires some understanding of the organisational context in which it was developed. The specific reference to ‘chickenpox’ - both in Amy’s description of her summarising role (Additional file 1: Box 1) and (again) in this short document (Additional file 1: Box 2) - is a legacy of previous organisational history. The summarisers *used to* record chickenpox as a ‘Minor’ problem, but when doctors had advised them to include *only* diagnoses which they regarded as ‘Significant’ in their summaries, the summarisers had responded by suggesting to doctors that in certain *particular* circumstances (e.g. pregnancy) chickenpox might indeed be significant. An agreement had therefore been reached that chickenpox be included in future summaries as a ‘Significant’ problem and it had been re-categorised as ‘Significant’ ever since. Its appearance in this document is not simply an ‘*overview of what summarisers do*’ but a reference to a small triumph of administrators over doctors in the organisation’s recent past.

In practice, a number of problems which summarisers might previously have defined as ‘Minor’ problems they now defined as ‘Significant’. This meant they could satisfy the requirement of the official summarisation protocol (which included: “*It is vitally important that nothing is missed*”), exercise their wish to be thorough, and also satisfy (by means of a workaround) the doctors’ request that problems which are important enough to be in the Summary should be ‘Significant’ ones. One of the summarisers justified this workaround further by pointing out that when doctors do home visits and take a ‘summary printout’ with them (a paper summary of the patient’s EPR), this printout lists only ‘significant’ problems, not ‘minor’ problems. Classifying problems as ‘minor’ might, she said, risk compromising care for patients in this situation. This is further evidence of the tension experienced by administrators between their formal accountability to GPs and the institution, and a sense of informal accountability to patients.

As part of the recent drive to get more summaries completed, one of the GPs had developed a paper form for his GP colleagues to complete as they selected items from the patient’s record for the Summary. There were separate sections for smears, past medical history (Significant Active and Significant Past), allergies, and immunisations. The section for ‘Immunisations’ said, in brackets alongside “(*if time permits, without this paper records have to be dug out if patient enquires*)*”*. This qualifying note suggests that ‘Immunisations’ were not only regarded by this GP as low priority compared to other parts of the record, but constructs meanings around the relative value of time for different members of staff. It is unlikely that lack of time would be a legitimate reason for an administrator to omit immunisations from a patient’s EPR. Indeed, administrators often entered details of all immunisations into the EPR first, before tackling other aspects, painstakingly copying vaccine batch numbers and entering codes for ‘place of procedure’ alongside, meticulous in their efforts to ensure complete immunisation records. Arguably an incomplete immunisations record would be more troublesome to nurses in their daily work than to GPs (who rarely give immunisations outside of the annual influenza campaign and rely on nurses to run the travel clinic). The labour intensive work of ‘*digging out*’ patients’ notes in the middle of a busy travel clinic is work that would likely fall to nurses and receptionists. This was one of several examples we encountered of different staff groups having different intentions and assumptions shaping what constitutes the summarised record [39].

The paper form that was part of the new summarising routine for GPs was an intermediate document. Here, GPs identified *what* they wanted to include in the summary, without investing any time identifying specific Read codes to capture the concept. This work of matching items on the list to appropriate Read codes was passed back to the administrators, in a move which may suggest that the GPs considered that the most important judgements lay in the *selection* of items for summarisation, rather than in the Read coding itself.

Amy gave each GP five sets of notes for summarising per week, keeping a record of who had been sent which notes on an Excel spreadsheet. GPs returned their completed forms to the summarisers, listing the items they wished to be entered into the patient’s EPR summary. The summarisers entered these items into the EPR one by one, choosing appropriate Read codes. Not all GPs had kept up with this workload, and whilst administrators said they were delighted that the GPs were helping them out, they were uncomfortable about the way the process was unfolding. There was concern that their protocol (Additional file 1: Box 2) was not being followed and that the GPs’ summaries were not sufficiently detailed. Some diagnoses were not being included and records of immunisations and cervical smears were sometimes incomplete (it is of note that the work of cervical screening is done almost entirely by nurses, who do the smears, and administrators who manage registration and recall). However, the administrators had (reluctantly) agreed that they would not do any further checking against the original medical notes (which would incur the very time penalties that this division of labour was supposed to address). They would simply enter Read codes for the selected items as the GPs requested. This was a source of significant tension for summarisers.

An integral part of the summarising routine was that a Read code (“*notes summary on computer*”) was entered into the patient’s EPR when a summary was completed. This ‘meta-data’ or ‘data about data’ was a QOF requirement and itself subject to regular audit. The administrators had discussed their concerns over different standards of summarisation with the practice manager, and as a result they had identified a *different* Read code (“*Lloyd George*^*a*^*and Problem Summary”*) which would be understood (locally) to mean that a *doctor* (rather than an administrator) had done the summary (this subtlety of meaning would be lost on transfer of records beyond the surgery). Administrators felt that this would cover them in the event of any queries, and for completeness they added a free text entry of the specific GP’s initials next to the Read code. The summarisers had constructed a particular notion of summarising which they cherished and which constituted their own ‘gold standard’. In this instance the EPR was being used resourcefully by administrators to facilitate surveillance of their employers (the GPs) in what seemed like a curious reversal of the usual lines of accountability.

The related organisational routines around Read coding the incoming post provided similar opportunity for contests around competing lines of accountability to be performed. Discharge letters and reports of outpatient clinic attendance received at Clover were scanned by a receptionist to produce an electronic document for circulation first to a GP and then to a coder using a document management system which was integrated with the EMIS system. The GPs read and highlighted the document electronically and decided whether any specific actions were needed, identifying these in comment boxes. Items for Read coding were highlighted in grey; a yellow highlighting function was used to make certain parts of the letters more visible to future readers. As with the summarising protocol, coders were to categorise Read codes as ‘Significant’. One GP liked to do most of his Read coding himself; another occasionally did. Most doctors relied on the coders.

Coding involved three broad dimensions of increasing complexity. The technical demands of EMIS and the document management system were relatively straightforward. Letters were re-directed, notes added, and workflows ‘terminated’ once letters were coded. Selecting Read codes which matched the grey highlighting was more troublesome and fraught with the same challenges that the summarisers encountered. Most difficult of all was managing the social complexities of this task, in a (virtual) environment in which each GP had their own preferred ways of working. A short session of coding could generate many queries to resolve (Additional file 1: Box 3).

An extract from DS’ reflective journal written just after this observation read:

*I was struck by the balancing act that I had been observing – by the coder’s clear sense that she was serving both patient and doctor and always trying to gauge their interests. I realised that coding a record is not the unproblematic technical task which it is so often assumed to be, but a highly social phenomenon and one which involves interpretation and judgement at so many levels. Deciphering poor handwriting; contradictory entries in notes; diagnoses that have no Read codes at all; Read codes which seem ‘not quite right’ for the particular problem. But most of all I realised how difficult it was to make those moral judgements about whether to act on (or quietly ignore) concerns that coding may not be perfect (but can it ever be?), whether and how to craft those notes to doctors, and how to gauge how different personalities in different and particular circumstances might react to receiving such notes. As she said, all the doctors are different and do things in different ways. Mastering the coding task was much less about coding and computers and so much more about managing relationships than I might ever have imagined. How a patient’s record is coded is not only (or even mainly) about ‘capturing’ and representing specific diagnoses as bytes of data, but is a product of complex and nuanced interactions between clinicians and administrators shaped not only by the ‘facts’ of the case, but the ongoing relationships which are co-constructed alongside the ‘problem list’.* [Extract from reflective journal]

Once medical judgements are no longer the unique province of the doctor, other staff members such as coders and summarisers have responsibilities which are not always socially recognised in the hierarchy of the surgery. On the one hand the GP’s authority is undermined by the potential for work to become more distributed. The EPR’s wide ‘organisational reach’ and its ready openness to surveillance by other members of the practice – such as administrators – opens up scope for the medical judgements of doctors to be scrutinised (and criticised) by them [40]. Conversely the GP’s authority within the social environment of the practice is carefully maintained (see Additional file 1: Box 3). This is in part constituted through the social actions of GPs, but more importantly it arises from the reciprocity of social actions and interactions between GPs and administrators which serves to maintain the social hierarchy. Amongst the many interpretive judgements that Linda had to make, the trickiest part of the coding role was around how to ‘act’ in situations where ‘no action’ was recommended by the GPs. ‘*No action*’ never meant that no action was taken by Linda. If anything, it was when ‘no action’ was recommended by doctors that the coding task became most perplexing. It was in these circumstances that different perspectives on what constituted a high quality summary came to the foreground and her moral sense of informal accountability towards patients jostled (and often jarred) with formal accountabilities towards the doctors and the organisation [37]. Adding to this complexity, the social negotiations which ensued were carried out primarily within virtual networks through written text, and remained visible to anyone who chose to study the ‘audit’ trail at a later date. The coder performed her identity as a competent, conscientious, caring worker trying to meet her informal obligation to patients. At the same time she was also creative in finding ways of respectfully engaging the GPs in a new kind of exchange where professional hierarchies and local accountabilities were being constantly renegotiated and notions of what constitutes high quality Read coding were being refined and revisited in every exchange. Linda was widely acknowledged to be particularly good at her job by GPs and management alike.

## Conclusions

It is often widely assumed that UK primary care data is recorded by clinicians and emerges from routine clinical care in a relatively ‘effortless’ fashion. By contrast it is widely recognised that hospital data recording is the province of data entry clerks. This paper not only highlights coding work as an *effortful* accomplishment, but identifies it as work which is often routinely shared with – or delegated to – administrative staff. The work of administrators summarising and coding records is complex, socially demanding and resource intensive. It is also work which provides an opportunity for administrators to contribute to new understandings of what constitutes a ‘quality’ summary and patient record. This contributes in an ongoing way to the construction of the local ‘information context’ and to particular norms of information management which are shaped through repeated iterations of coding and summarising routines. In parallel with this, there is space for new lines of accountability to be negotiated.

The simplest but most striking observation is the extent to which the EPR is ‘attended to’ or ‘cared for’ throughout the organisation by doctors and administrators alike. It may not be surprising that such labour-intensive work is referred to using anthropomorphic terms such as ‘feeding’ and ‘toileting’. Also striking is the ‘taken-for-granted’ nature of this work. Despite well recognised concerns from doctors that administrators may be paying too much attention to detail in their coding practices and an understanding by coders that the doctors want something more ‘basic’, the administrators were developing their status as local ‘experts’.

For example, Linda was sufficiently trusted as an expert in coding and summarising that one GP had (informally) agreed with her that she may add Read codes to EPRs as she felt appropriate, and GPs regularly commented on the high quality of her work. In addition, administrators took ownership of the implementation of the electronic record transfer system, making a collective decision to ‘start over’ with summarising in order to maintain their own particular standards. Their redesign of work practices by producing a simplified summarisation ‘protocol’ for doctors (Additional file 1: Box 2) and their identification of doctors’ summaries with a new Read code to distinguish doctors’ work (which they perceived as poorer in quality) from their own was further evidence of their reflective and critical stance towards this important work. This attention to ongoing reflection and work redesign has been identified by researchers investigating the working practices of coders in litigation [22,23] and we have described parallels in the work of reception staff in general practice as they seek to uphold the quality and safety of repeat prescribing [37]. Linda often re-routed letters back to GPs, and was supported by them in this practice when she had concerns that some ‘no action’ items may need further attention, even though this was clearly a sensitive task which required careful handling and involved a certain risk to ‘self’ as she engaged in it.

Administrators kept in the balance two different ‘versions’ of the patient, the patient as individual ‘person’, to whom they felt informally accountable, and the patient as one of a population of patients sharing some characteristic of institutional relevance (a manifestation of what we have termed a ‘dilemma of attention’) [36,41]. In parallel they also had to manage what were sometimes competing perspectives on the purpose of coded entries in the EPR, and a moral tension between their idealised aspiration of a ‘gold standard’ record and a pragmatic recognition that this is rarely achievable in practice. This tension became particularly evident when GPs (to whom they were formally accountable) started assisting with summarising records. This became an opportunity for redefining lines of accountability as administrators distanced themselves from what they perceived as inferior coding practices and ensured that a unique Read code was identified to distinguish doctors’ coding from their own. Similar tensions occurred in the coding of incoming post and in the delicate acts of negotiation between coder and GP when disagreements arose over what constituted adequate Read coding of letters.

Although administrators were able to work creatively with the EPR in ways which *challenged* existing organisational hierarchies and constructed new lines of accountability, the exercise of this ‘accountability work’ was highly mediated and hedged, emotionally laden, and (on the whole) operated within constraints which favoured the maintenance of the social order in the hierarchy, even as work was being distributed and responsibilities shared. However, within every exchange between administrator and clinician around coding and summarising lay a small opportunity for adjustments to the social hierarchy and the possibility that the strength of influence of staff in the ‘back office’ might grow over time.

The tendency was for summaries at Clover to get longer and more detailed through these administrative processes, despite the widespread understanding that ‘*cluttered*’ screens were best avoided. As summaries get longer, so the potential develops that someone somewhere else in the organisation may edit or ‘*clean up*’ the EPR, as yet further effort is invested in ‘*caring*’ for it. This collective attention throughout the organisation to producing, maintaining and editing the EPR – quite apart from the equipment and technical support that is required to keep the EPR operational – constitutes the EPR as significant and central to practice life. This ‘meaning-making’ around the EPR is constructed and sustained through repeated small and seemingly mundane moment-by-moment practices of organisational actors as they engage with the EPR, and with each other around the EPR.

Clover Surgery, as a ‘pro-technology’ case study site was a particularly well-developed example of the work of summarising and coding records. It is because clinicians, administrative and management staff were so conscientious in their efforts to do this work well that we have been able to elucidate the theoretical insights presented in this paper. We cannot make any generalisable claims about the details of coding and summarising work in other surgeries as this is likely to be operationalised in different ways in different contexts. However, we suggest that the kinds of opportunities, challenges, dilemmas and tensions that we have seen played out in our case study site, and the interdependencies between coding practices, inter-professional working relationships, institutional norms and wider socio-political context are likely to be critical to the ‘quality’ of patient data more widely. Administrative staff make substantial, time-consuming, and largely ‘hidden’ contributions to the quality of patient data, considering themselves accountable to patients as well as doctors for the quality of this work and making ongoing nuanced moral judgements about how best to act in situations of uncertainty. Current interest in large datasets and the potential for health data to be put to an ever widening array of secondary uses tends to obscure the socially complex work that lies in the details of how data gets onto the record, and we suggest that this presents an important, often overlooked agenda for research on the quality of health care.

## Endnote

^a^Lloyd George is a reference to standard record cards (and envelopes) that UK NHS GPs used to document and store their medical notes before electronic records were widely used.

## Additional file

Additional file 1:
**Box 1.** Field notes on summarising (Clover Surgery). **Box 2.** Extract of document prepared for GPs by summarisers at Clover Surgery. **Box 3.** Fieldnotes on coding incoming post, Clover (original notes edited for brevity).

## References

[1] Majeed A, Car J, Sheikh A (2008). Accuracy and completeness of electronic patient records in primary care. Fam Pract.

[2] Thiru K, Hassey A, Sullivan F (2003). Systematic review of scope and quality of electronic patient record data in primary care. BMJ.

[3] de Lusignan S (2006). The optimum granularity for coding diagnostic data in primary care: report of a workshop of the EFMI Primary Care Informatics Working Group at MIE 2005. Inform Prim Care.

[4] de Lusignan S, van Weel C (2006). The use of routinely collected computer data for research in primary care: opportunities and challenges. Fam Pract.

[5] Jordan K, Porcheret M, Croft P (2004). Quality of morbidity coding in general practice computerized medial records: a systematic review. Fam Pract.

[6] NHS. Your records: Better information means better care. NHS Choices. http://www.nhs.uk/NHSEngland/thenhs/records/healthrecords/Pages/care-data.aspx. 2013.

[7] de Lusignan S, Wells SE, Hague NJ, Thiru K (2003). Managers see the problems associated with coding clinical data as a technical issue whilst clinicians also see cultural barriers. Methods Inf Med.

[8] Lusignan D (2005). The barriers to clinical coding in general practice: a literature review. Inform Health Soc Care.

[9] Department of Health, RCGP, BMA: The Good Practice Guidelines for GP electronic patient records v4. Downloadable from: http://www.dh.gov.uk/prod_consum_dh/groups/dh_digitalassets/documents/digitalasset/dh_125350.pdf. 2011.

[10] Greenhalgh T, Keen J (2013). England's national programme for IT: From contested success claims to exaggerated reports of its death. BMJ.

[11] Department of Health: Liberating the NHS: An Information Revolution. Downloadable from http://webarchive.nationalarchives.gov.uk/20130107105354/http://www.dh.gov.uk/en/Consultations/Liveconsultations/DH_120080. 2010.

[12] Emmerich N, Swinglehurst D, Maybin J, Park S, Quilligan S. Caring for Quality of Care: Symbolic Violence and the Bureaucracies of Audit. BMC Medical Ethics. 2015; http://dx.doi.org/10.1186/s12910-015-0006-z. 10.1186/s12910-015-0006-z PMC4493962 25952931

[13] Health and Social Care Information Centre. Read Coded Clinical Terms. See: http://www.datadictionary.nhs.uk/web_site_content/supporting_information/clinical_coding/read_coded_clinical_terms.asp?shownav=1. 2013. Health and Social Care Information Centre.

[14] Health and Social Care Information Centre (2013). Sandards for the clinical structure and content of patient records.

[15] Heeks R, Mundy D, Salazar A. Why Health Care Information Systems Succeed or Fail. 9. 1999. University of Manchester, Institute for Development Policy and Management. Information Systems for Public Sector Management Working Paper Series.

[16] Berg M (2004). Health care work and patient care information systems. Health Information Management - integrating information technology in health care work.

[17] Bowker G, Star S (2000). Sorting things Out: classification and its consequences.

[18] Star SL (1995). Ecologies of knowledge.

[19] Berg M (1996). Practices of reading and writing: the constitutive role of the patient record in medical work. Sociol Health Illness.

[20] Berg M (1997). Of Forms, containers, and the electronic medical record: some tools for a sociology of the formal. Sci Technol Hum Values.

[21] Suchman L (2002). Located accountabilities in technology production. Scand J Inf Syst.

[22] Blomberg J, Suchman L, Trigg RH (1996). Reflections on a work-oriented design project. Hum Comp Interact.

[23] Suchman L (1995). Making work visible. Commun ACM.

[24] Swinglehurst D, Greenhalgh T, Roberts C (2012). Computer templates in chronic disease management: ethnographic case study in general practice. BMJ Open.

[25] Greenhalgh T, Swinglehurst D (2011). Studying technology use as social practice: the untapped potential of ethnography. BMC Med.

[26] Swinglehurst D, Greenhalgh T, Myall M, Russell J (2010). Ethnographic study of ICT-supported collaborative work routines in general practice. BMC Health Serv Res.

[27] Pope C (2005). Conducting ethnography in medical settings. Med Educ.

[28] Stake RE: Qualitative Case Studies. In The Sage Handbook of Qualitative Research. 3rd edition. Edited by Denzin NK, Lincoln YS. Sage Publications, Inc; 2005:443–466.

[29] Hammersley M, Atkinson P (1995). Access. Ethnography: principles in practice.

[30] Barley S, Tolbert P (1997). Institutionalization and structuration: studying the links between action and institution. Organ Stud.

[31] Rose G (1982). Deciphering Sociological Research.

[32] Geertz C (1973). Thick description: toward an interpretive theory of culture. The interpretation of cultures.

[33] Ormrod S (2003). Organisational culture in health services policy and research: 'third way' political fad or policy development?. Policy Politics.

[34] Goffman E (1959). Regions and region behaviour. The presentation of self in everyday life.

[35] Mitchell J, Ellen R (1984). Producing data: case studies. Ethnographic research: a guide to general conduct.

[36] Swinglehurst D (2014). Displays of authority in the clinical consultation: a linguistic ethnographic study of the electronic patietn record. Soc Sci Med.

[37] Swinglehurst D, Greenhalgh T, Russell J, Myall M (2011). Receptionist input to quality and safety in repeat prescribing in UK general practice: ethnographic case study. BMJ.

[38] Robinson JD (1998). Getting down to business: talk, gaze and body orientation during openings of doctor-patient consultations. Hum Commun Res.

[39] Cochran N, Gordon AC, Krause MS (1980). Proactive records. Knowledge.

[40] Iedema R (2003). The medical record as organizing discourse. Doc Des.

[41] Swinglehurst D, Roberts C, Greenhalgh T (2011). Opening up the "black box" of the electronic patient record: A linguistic ethnographic study in general practice. Commun Med.

